# A commentary of “the intersection of archaeology and genomics: Sparking the advances in cognitive human society”: 10 remarkable discoveries from 2020 in *Nature*

**DOI:** 10.1016/j.fmre.2022.01.011

**Published:** 2022-01-30

**Authors:** Kai Ye

**Affiliations:** MOE Key Lab for Intelligent Networks and Networks Security, Faculty of Electronic and Information Engineering, Xi'an Jiaotong University, Xi'an, Shaanxi, China

## Abstract

Cassidy et al. of Trinity College in Dublin, Ireland, studied the social structure of farming communities, focusing on the ancient nobles buried in passage tombs (a channel-style megalithic tomb structure in Europe). Newgrange, built by complex engineering technology, is the most famous stone tunnel tomb in Ireland and one of the most famous prehistoric cemeteries in the country. The tomb is at the end of a stone-lined passage, with an opening like a window above the mausoleum entrance. On the shortest day of the year (winter solstice), this opening allows sunlight to enter the tomb. Researchers conducted DNA analysis of ancient human remains found in the tomb, revealing a rare and unexpected incident of incest. A man buried in Newgrange's tomb about 5000 years ago was the offspring of an incestuous marriage: his parents were either siblings or parents and children. This discovery led the researchers to speculate that the nobles associated with this magnificent tomb may have maintained their bloodlines through incest.①

Since ancient times, human beings have never given up on the study of ourselves. Throughout distinct periods, different regions of the world have shown diverse social characteristics according to the status of the times. The formation and evolution of social class patterns is consistently a hot topic of human research. Due to the lack of technology for the long-term preservation of text or images from the initial stages of human society, archaeological experts generally use specific archeological methods to understand and excavate early social formation, morphological development, historical trajectories, and social characteristics. In 2020, archaeologists and genomics experts adopted whole-genome sequencing methods to better understand the distribution and inheritance of political power in the Neolithic Age in Europe. They used genome sequencing technology to analyze 44 remains in the tomb of Newgrange, Ireland, revealing that a man buried 5000 years ago was the offspring of first-degree incest; that is, his parents are either brothers and sisters, mother and son, or father and daughter [1]. Researchers also found the relatives of the man in a tomb 150 kilometers away, revealing that in the process of large-scale Neolithic marine colonization to replace small-scale scattered tribes in the Mesolithic era, ruling class elites emerged and adopted a hereditary system to maintain the long-term role of the specific families. In earlier stages, when human society had not yet developed a more advanced system to dynamically adjust the ruling class, incest in a small number of ruling families, to give birth to pure offspring could stabilize classes and legitimize the rule of power. This research elegantly uses genome sequencing technology to analyze and compare Neolithic genotypes and dietary structures across Europe, revealing formation evidence of early ruling classes in human society and was selected by Nature as one of the “Top Ten Scientific Discoveries” in 2020. In addition to the significance of this research to human social cognition, the strengths of multidisciplinary and joint research and innovative interpretation shown in the article are very exciting in today's scientific research environment dominated by big data.

This research uses genomics to make breakthroughs in human social cognition, referred to as social cognitive genomics. The following directions will likely be explored in-depth in the future: (1) Multi-regional joint analysis, conducted by an international alliance on a global scale analyze and compare the evolution of human society in different regions during the same period and incorporate those of animal and plant to reveal the interaction of the environment, humans, and society in specific regions. Due to advanced archaeological research in Europe, this paper combines innovative genome sequencing technology to reveal the early characteristics of human society for the first time. However, in other parts of the world, especially in Asia, the birthplace of world civilization, data is very limited, hindering our understanding of the evolution of human society on a global scale. (2) Comparative analysis at multiple time points will be used in the future to determine the ages of each sample in a more detailed manner, comparing the environmental, human, and social characteristics of the same region in more detail, albeit at different ages, revealing the law of human social changes before and after. (3) Multi-region and multi-time point analysis will be used to study the evolution of global human society as a dynamic development as a whole, revealing the spatiotemporal relationship between the gene flow in the process of human migration, the gene flow of animals and plants that accompany human migration or trade, and the social "gene" flow of religion and organizational forms.

China's archaeology field has significant advantages owing to thousands of years of historical civilization development. As such, although the field of genome research is also very active, there is a lack of interaction between two original research disciplines demonstrated in this study due to the missing environment enabling interdisciplinary researches. This is simply due to the fact that archeologists want more detailed archaeological evidence, relying on the analysis results of genomic research experts of relevant original genetic data. The analysis results of genome experts also require the expertise of archaeologists to reveal scientific value or practical significance. The actual issue is that in the current era of the knowledge explosion, the producers, and owners of big data in any field in China are often top experts in one field, not generalists proficient in various fields. Researchers who have the capability to discover the most significant data value are often not the ones to obtain the data or are unaware of its existence. How to motivate Chinese scholars to organize multidisciplinary teams to generate data and to interpret data in depth under the situation of increasingly fierce global scientific and technological competition and significant interdisciplinary advantages is a problem that Chinese scientific research management departments and scholars of various disciplines need to resolve jointly.

## Declaration of Competing Interest

The author declares that there is no conflict of interest in this work.
